# Spontaneous bacterial peritonitis complicating extensive splanchnic vein thrombosis, a rare manifestation of essential thrombocythemia: A case report

**DOI:** 10.1002/ccr3.7634

**Published:** 2023-07-02

**Authors:** Saurav Agrawal, Sandesh Rayamajhi, Aadesh Rayamajhi, Susmin Karki, Anand Deo, Kirti Kala Kharel, Manoj Lamsal, Rabin Hamal

**Affiliations:** ^1^ Maharajgunj Medical Campus, Institute of Medicine Tribhuvan University Kathmandu Nepal; ^2^ Nepalese Army Institute of Health Sciences College of Medicine Kathmandu Nepal; ^3^ Department of Internal Medicine Tribhuvan University Teaching Hospital Kathmandu Nepal; ^4^ Department of Internal Medicine Nobel Medical College Biratnagar Nepal; ^5^ Department of Gastroenterology Tribhuvan University Teaching Hospital Kathmandu Nepal

**Keywords:** ascites, case report, myeloproliferative neoplasm, peritonitis, thrombocythemia, thrombosis

## Abstract

**Key Clinical Message:**

Clinicians should be mindful of the rare occurrence of spontaneous bacterial peritonitis in essential thrombocythemia with extensive splanchnic vein thrombosis, especially when patients with ascites exhibit fever and abdominal pain.

**Abstract:**

Spontaneous bacterial peritonitis (SBP) complicating extensive splanchnic vein thrombosis (SVT) is a rare manifestation of essential thrombocythemia (ET). In the absence of any hypercoagulable state, JAK2 mutation can be an important risk factor for extensive SVT. Evaluation for SBP is crucial when non‐cirrhotic patient exhibits fever, abdominal pain and tenderness in the background of ascites after ruling out common pathologies such as tubercular peritonitis, acute pancreatitis, Budd‐Chiari syndrome and ovarian malignancy. We present a case of SBP complicating pre‐hepatic portal hypertension with ascites in a 44‐years‐old female. On further evaluation, extensive SVT with portal cavernoma in the setting of ET was identified. She was managed with cytoreductive therapy and anticoagulation, resulting in symptom resolution.

## Graphical abstract

Extensive splanchnic vein thrombosis with a completely occluding thrombus in the main portal vein, superior mesenteric vein and splenic vein precipitated spontaneous bacterial peritonitis in a non‐cirrhotic 44‐year‐old female with JAK2 V617 positive essential thrombocythemia.

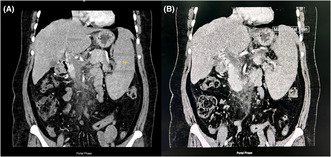

## 1 INTRODUCTION

Splanchnic vein thrombosis (SVT) represents a distinct manifestation of venous thromboembolism involving one or more abdominal vessels such as portal vein, mesenteric vein, splenic vein and hepatic veins.^1^ SVT is infrequently observed in the general population, with portal vein thrombosis (PVT) and mesenteric vein thrombosis (MVT) occurring at a rate of 0.7 per 100,000 and 2.7 per 100,000, respectively.^2^ Cirrhosis, recent abdominal surgery, inflammatory bowel disease, intra‐abdominal infection, solid malignancies, and pancreatitis are among the most frequently observed triggering factors.^3^ In the absence of cirrhosis or neighboring malignancies, myeloproliferative neoplasms (MPN) are the primary cause of SVT.^4^

In case of chronic PVT, obstructed portal vein is bypassed by a network of portoportal collateral veins called as portal cavernoma. However, these collaterals are not sufficient to normalize hepatopetal blood flow and hence eventually portal hypertension develops.^5^ Overtime, portal hypertension may give rise to severe complications such as variceal bleeding, ascites, or hepatic encephalopathy.^4^ Spontaneous bacterial peritonitis (SBP) complicating pre‐hepatic portal hypertension is extremely rare and limited to only few case reports in the literature.^6^

Herein, we present a case of SBP complicating pre‐hepatic portal hypertension with ascites in a 44‐year‐old female in the setting of extensive chronic SVT secondary to essential thrombocythemia (ET).

## 2 CASE PRESENTATION

A 44‐year‐old Nepali female, known case of systemic hypertension, presented to emergency department (ED) with complaints of abdominal distension for 1 month and fever with generalized abdominal pain for 7 days. The pain was continuous, dull aching, moderate in intensity, associated with multiple episodes of non‐bloody vomiting. There was no history of diarrhea, melena or jaundice and no any other history suggestive of chronic liver disease (CLD).

Upon presentation to ED, the patient was conscious, oriented with pulse rate of 112 beats per minute, blood pressure of 130/90 mm Hg, respiratory rate of 20 breaths per minute with 98% saturation on room air and fever of 101 F. Abdominal examination revealed a distended abdomen with tenderness present over bilateral hypochondriac and epigastric regions. Spleen was palpable 3 cm below the left costal margin. Examination of other systems was unremarkable. Based on the notable clinical manifestations, differential diagnoses were formulated encompassing liver cirrhosis, severe acute pancreatitis, Budd‐Chiari syndrome (BCS), and complicated ovarian malignancy. Tubercular peritonitis was also kept into consideration based on endemicity of the pathogen.

**FIGURE 1:**
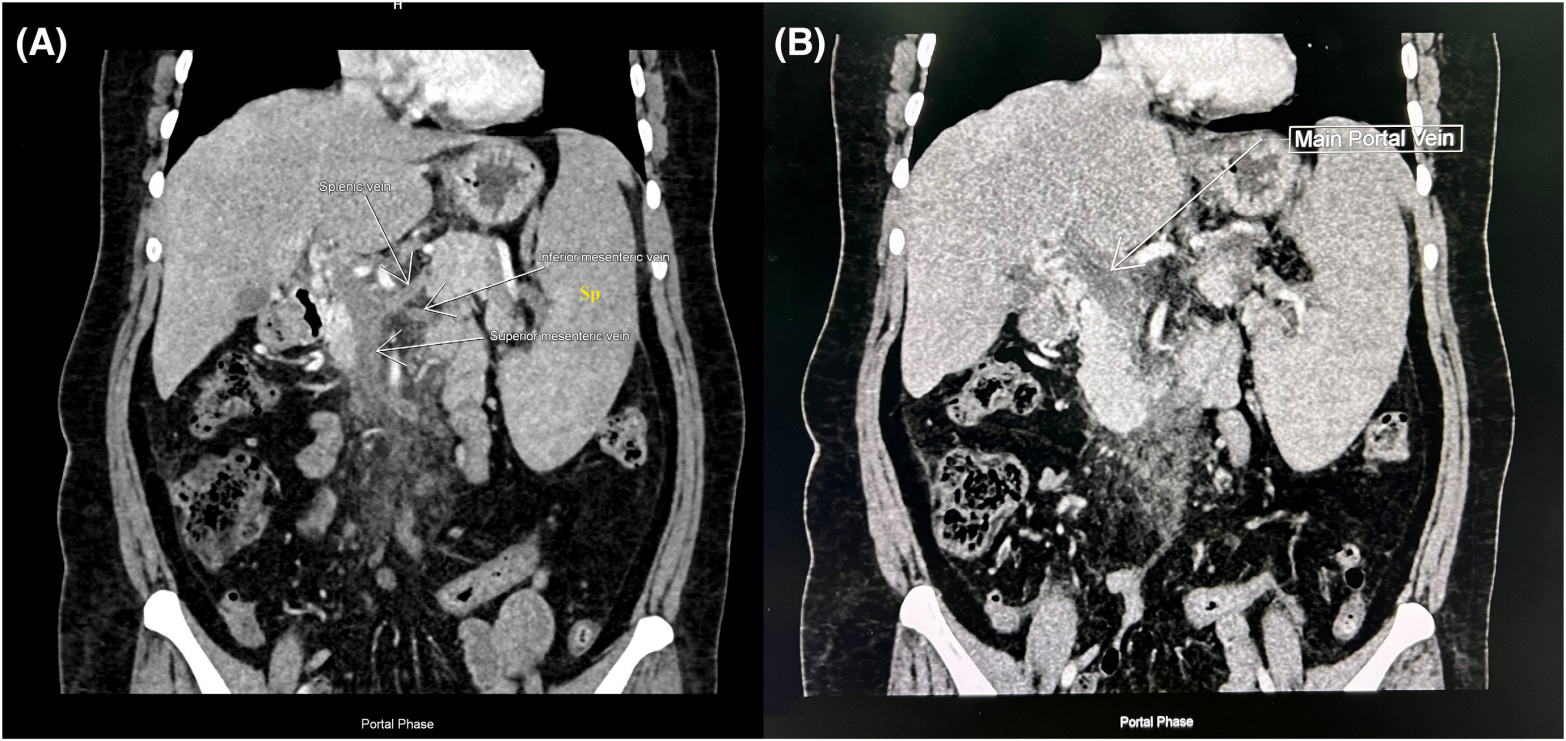
CECT of coronal section of abdomen showing. (A) Enlarged spleen (Sp) with completely occluding thrombus in superior mesenteric vein and splenic vein (arrows) in the portal phase. (B) Thrombus in main portal vein in the portal phase.

**FIGURE 2:**
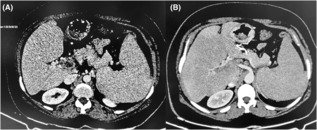
CECT of axial section of abdomen showing multiple collaterals vessels in (A) Peri‐portal region. (B) Peri‐gastric region.

A complete blood count revealed leukocytosis with total leukocyte count (TLC) of 16,500/mm^3^, marked thrombocytosis with platelets of 729,000/mm^3^, a hemoglobin of 11.2gm% and hematocrit of 37%. Peripheral blood film examination revealed normocytic normochromic red blood cells with neutrophilic leukocytosis and thrombocytosis. Erythrocyte sedimentation rate, blood glucose, renal function tests, liver function tests and iron profile were all within normal limits. Ultrasonography of the abdomen showed normal liver parenchyma, moderate splenomegaly (16 cm), moderate ascites with non‐visualization of portal vein, features suggestive of portal vein thrombosis which was confirmed with contrast‐enhanced computed tomography (CECT) scan of the abdomen and pelvis that revealed splenomegaly with a completely occluding thrombus in the main portal vein, right and left portal vein, superior mesenteric vein and splenic vein (Figure 1). There were also multiple collaterals vessels in peri‐portal, peri‐gastric and splenic hilar region confirming the presence of portal cavernoma secondary to chronic splanchnic venous thrombosis (Figure 2). The absence of stigmata of CLD on examination, along with normal liver chemistries and serology, and normal liver parenchyma and vasculature on imaging ruled out conditions like liver cirrhosis and BCS. In addition, the serum levels of amylase and lipase were within the normal range, and the pancreatic morphology appeared unaffected in the CECT scan. Moreover, the imaging results revealed no presence of pelvic masses, such as ovarian cysts, which could potentially undergo torsion and cause pain in female patients with ascites. Esophagogastroduodenoscopy of the patient was normal. Ascitic fluid analysis revealed a TLC of 2200 cells/mm^3^, polymorphonuclear cell count of 1760 cells/mm^3^, with ascitic fluid protein of 3 g/dL, albumin of 1.8 g/dL, serum ascites albumin gradient of 2.1 g/dL with normal adenosine deaminase level. Culture of ascitic fluid yielded confluent growth of *Klebsiella pneumoniae*. Consequently, the tubercular peritonitis was excluded from differentials due to the absence of lymphocytic predominance and normal adenosine deaminase levels in the ascitic fluid with absence of peritoneal, intestinal and lymph node changes on CECT.

**FIGURE 3:**
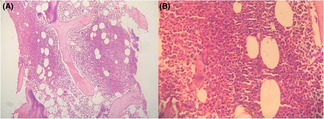
Histopathology showing hypercellular marrow with active erythropoiesis and prominent megakaryocytic hyperplasia in (A) 100× magnification. (B) 400× magnification

The patient was admitted to an intensive care unit and managed with intravenous fluids, bowel rest and empirical antibiotics therapy followed by specific antibiotic as per culture and sensitivity report. There were no notable findings during the evaluation for thrombophilia, including factor V Leiden, prothrombin gene mutations, protein C, protein S, antithrombin III, and antiphospholipid antibodies. In view of extensive SVT involvement, she was also tested for lupus anticoagulant, anticardiolipin antibody isotype immunoglobulin (Ig)G, IgM, β2 glycoprotein IgG, IgM, which came out to be negative. Additionally, there were no acquired risk factors for thrombosis such as prolonged immobilization, trauma, or intra‐abdominal inflammatory processes. With no secondary cause apparent for persistent thrombocytosis, hematology consultation was done. Subsequently a molecular study revealed JAK2 V617F mutation positivity and was negative for myeloproliferative leukemia (MPL), and calreticulin (CALR) gene mutations. This prompted a bone marrow biopsy which showed hypercellular marrow with active erythropoiesis and prominent megakaryocytic hyperplasia consistent with essential thrombocythemia (Figure 3). All the WHO 2016 diagnostic criteria for ET were fulfilled.

With the diagnosis of SBP complicating pre‐hepatic portal hypertension secondary to JAK 2 positive ET, patient was started on cytoreductive therapy (hydroxyurea) under close hematological monitoring. SVT was managed with heparin followed by warfarin with target international normalized ratio of 2–3, monitored daily during hospital stay. After these events, there was gradual resolution of her symptoms and ascites during her 15 days of hospital stay with platelet of 167,000/mm^3^, TLC of 3400/mm^3^. She was discharged with hydroxyurea for her hematological disease and anticoagulation with warfarin as a prophylaxis for recurrent thrombosis.

## 3 DISCUSSION

Essential thrombocythemia (ET) is recognized as a myeloproliferative neoplasm (MPN), a condition marked by an upsurge in platelet counts and a profusion of morphologically mature megakaryocytes found in the bone marrow.^7^ With an estimated annual incidence of 0.6–2.5 cases per 100,000, ET has a comparatively favorable prognosis when compared to other MPNs.^8^, ^9^ The mean age at diagnosis for ET is 58 years and tends to occur predominantly in the adult female population.^10^ Most individuals display Janus activated kinase 2 (JAK2) V617F variant, calreticulin (CALR), or myeloproliferative leukemia (MPL) mutations (60%, 20%, and 3%, respectively), while 10% to 20% of ET cases do not exhibit any of these mutations (triple‐negative).^10^

Clinical presentations of ET can vary from vaso‐occlusive events and bleeding manifestations to later‐stage progression into leukemia or other types of myeloid neoplasms. Headache (27.5%) and abdominal or bone pain (5.5%) are the most common symptoms, however, half of the patients do not show any symptoms.^11^ For a diagnosis of ET to be made, all four major criteria or the first three major criteria and the minor criterion need to be met, which are as follows:

1. Major criteria: platelet count of 450 × 10^9^/L or higher, megakaryocyte proliferation and loose clusters observed in bone marrow, non‐conformity to WHO criteria for other myeloid neoplasms, presence of JAK2/CALR/MPL mutations
2. Minor criterion: presence of another clonal marker or the absence of evidence for reactive thrombocytosis.^12^
  Our patient had fulfilled all the major criteria for ET as defined by WHO.

At the time of diagnosis, ET affects major vessels and the microcirculation, resulting in thrombotic events that are observed in 20.7% of patients.^13^ Thrombosis has a predilection for targeting the central nervous system, the circulation of the heart, and the deep veins of lower extremities.^13^ While SVT is not the most common presentation of MPNs, it can occur in a minority of patients.^14^ Factors that increase the risk of MPN‐associated SVT include young age, female gender, concomitant hypercoagulable disorders, and the presence of JAK2 V617F mutation.^15^ Our patient was a female who carried the JAK2 mutation that might have predisposed her to develop SVT.

With the recent advancement in imaging, there has been timely and accurate diagnosis of SVT and its associated complications. CECT and imaging in the portal phase is considered as most reliable and accurate imaging modality as it can visualize both the extent of thrombosis within the portomesenteric venous system and secondary abnormal intestinal findings.^16^ In our case, CECT abdomen findings were suggestive of chronic SVT with multiple collateral vessels seen in periportal, perigastric and splenic hilar region.

A retrospective analysis of 181 patients with MPN‐related SVT revealed that 60.3% of them had a thrombus in the portal vein, while 17.1%, 13%, and 10% had thrombi in the hepatic vein, splenic vein, and mesenteric vein, respectively.^2^ Although it is uncommon for thrombus extension to affect more than one of the main splanchnic veins, SVT involving multiple veins has been rarely reported as the initial manifestation of ET, as in our case.^17^, ^18^, ^19^ Due to the absence of any other hypercoagulable states that could explain the presence of an extensive thrombus in our patient, JAK2 mutation can be regarded as a crucial prothrombotic risk factor for extensive SVT.

Spontaneous Bacterial Peritonitis (SBP) is an infection in the peritoneum that develops in patients with ascites related to cirrhosis.^20^ Gut dysbiosis and intestinal permeability in cirrhosis favor translocation of enteric bacteria from gut to mesenteric lymph nodes and eventually into ascitic fluid. This combined with impaired host defense systems in cirrhotic patients, including compromised neutrophil function and deficiencies in complement and opsonin, raises the risk of SBP. SBP can present in patient with cirrhosis as fever (69%), abdominal pain (59%), altered mental status (54%), abdominal tenderness (49%), diarrhea (32%), ileus (30%), hypotension (21%) or hypothermia (17%). It can be asymptomatic in 10% of cases especially related to large volume ascites.^21^ Based on presence of these symptoms and signs with concomitant ascites, SBP was suspected in our patient and she underwent diagnostic paracentesis. Diagnosis of SBP was confirmed based on positive ascitic fluid culture and a neutrophil count greater than 250/mm^3^.^20^

Instances of SBP are infrequent in extrahepatic portal vein obstruction (EHPVO) due to the combination of a low incidence of ascites, an intact hepatic reticuloendothelial system, and high protein content in the ascitic fluid.^6^ Similarly, patients with SVT can present with transient ascites which may get infected as was seen in this case. Therefore, a patient of ET presenting with abdominal pain in setting of ascites should also be evaluated for SBP as it is an uncommon manifestation in EHPVO and might masquerade as other causes of abdominal pain in ET such as splenic infarction and mesenteric ischemia.^17^, ^22^

## 4 CONCLUSION

SBP complicating chronic extensive SVT is a rare occurrence in essential thrombocythemia. Although there is well established association between JAK2 positive MPN and thrombotic events, pre‐hepatic portal hypertension presenting with complications such as ascites, SBP and splenomegaly are infrequently evaluated for JAK2 mutations. Therefore, molecular study and imaging modality can be valuable tools for the early detection of underlying pathology before presenting with such severe complications.

## AUTHOR CONTRIBUTIONS

**Saurav Agrawal:** Conceptualization; writing – original draft; writing – review and editing. **Sandesh Rayamajhi:** Conceptualization; writing – original draft; writing – review and editing. **Aadesh Rayamajhi:** Conceptualization; writing – original draft; writing – review and editing. **Susmin Karki:** Writing – review and editing. **Anand Deo:** Writing – review and editing. **Kirti Kala Kharel:** Writing – review and editing. **Manoj Lamsal:** Writing – review and editing. **Rabin Hamal:** Writing – review and editing.

## FUNDING INFORMATION

No external funding was received.

## CONFLICT OF INTEREST STATEMENT

The authors declare that there is no conflict of interest regarding the publication of this paper.

## ETHICS APPROVAL AND CONSENT TO PARTICIPATE

Need for ethical approval waived. Consent from the patient deemed to be enough.

## CONSENT

Written informed consent was obtained from the patient for publication of this case report and any accompanying images. A copy of the written consent will be available for review if asked by the editor‐in‐chief of this journal.

## Data Availability

Not applicable.
